# Canine Gastrinoma with Long-Term Survival After Primary and Repeat Surgeries Using Serial Serum Gastrin Concentrations as an Adjunctive Monitoring Marker: A Report of Two Cases

**DOI:** 10.3390/vetsci13070715

**Published:** 2026-07-20

**Authors:** Kyosuke Takeuchi, Kenji Hosoya, Ryo Owaki, Ryohei Kinoshita, Sangho Kim, Masahiro Okumura

**Affiliations:** 1 Veterinary Teaching Hospital, Hokkaido University, Kita 18 Nishi 9, Kita-ku, Sapporo 060-0818, Hokkaido, Japan; kyosuke.cham@bell.ocn.ne.jp (K.T.);; 2 PETEMO Animal Medical Center Sapporo Tsukisamu, BRANCH Sapporo Tsukisamu, 1-22, Tsukisamu-Higashi 3-jo 11-Chome, Toyohira-ku, Sapporo 062-0053, Hokkaido, Japan; 3 Laboratory of Veterinary Surgery, Department of Clinical Sciences, Graduate School of Veterinary Medicine, Hokkaido University, Kita 18 Nishi 9, Kita-ku, Sapporo 060-0818, Hokkaido, Japan

**Keywords:** computed tomography, dogs, gastrinoma, proton pump inhibitor, serum gastrin concentrations, surgery

## Abstract

Gastrinoma is a rare neuroendocrine tumor in dogs that arises from functional pancreatic non-β cells and secretes excessive gastrin. Gastrinoma in dogs has a high metastasis rate at diagnosis, and curative treatment is often difficult. There are few reports describing the long-term outcomes of surgical treatment in dogs with gastrinoma, and no established method for monitoring disease progression has been reported. We report here the cases of two adult dogs with pancreatic gastrinoma treated with surgical resection. Pharmacological therapy using proton pump inhibitors was administered to control gastrointestinal symptoms caused by hypergastrinemia. Multiple surgeries were performed to reduce the volume of primary and metastatic gastrinoma lesions. Serial serum gastrin concentrations were used as an adjunctive monitoring marker during follow-up. Changes in serum gastrin concentrations were used to support decisions regarding further imaging. Additional surgery was performed based on serum gastrin concentrations, clinical signs, and diagnostic imaging findings. A total of four surgeries were performed in Case 1 and three surgeries in Case 2. Both dogs maintained well-controlled gastrointestinal symptoms and showed long-term survival. The changes in serum gastrin concentrations in these cases suggest that serial serum gastrin measurement may be useful for monitoring recurrence or tumor progression in canine gastrinoma.

## Simple Summary

Gastrinomas are pancreatic non-β-cell tumors that secrete excessive gastrin and metastasize at a high rate; they occur rarely in dogs. While it is known that gastrointestinal signs caused by gastric acid hypersecretion can be controlled with the use of proton pump inhibitors, reports of surgical treatment are scarce and no established method for monitoring disease progression has been reported. In this study, serial serum gastrin concentrations were used as an adjunctive monitoring marker during follow-up. Computed tomography was used to evaluate the extent and distribution of lesions and to plan surgical treatment, and surgery was the only tumor-directed treatment performed. Both cases maintained good quality of life and achieved long-term survival after surgery.

## 1. Introduction

Gastrinoma is a functional neuroendocrine tumor that is rare in dogs, characterized by oversecretion of gastrin. Canine gastrinoma mostly originates in the pancreas, and the resulting hypergastrinemia causes refractory peptic ulcers, chronic diarrhea, esophagitis, and other gastrointestinal symptoms [1,2]. Gastrinoma has a high rate of metastasis in locations such as the lymph nodes, mesentery, peritoneal surface, and liver; metastases are often observed at initial diagnosis, making curative treatment often difficult [1,2,3,4].

Diagnosis of gastrinoma is generally aided by clinical signs, the presence of ulcer lesions, and markedly elevated fasting serum gastrin concentrations. Serum gastrin concentrations exceeding three to ten times the assay-specific upper reference limit (URL) have been suggested to support a diagnosis of gastrinoma in dogs [5]. Provocative testing or gastric pH measurements are rarely performed in veterinary medicine [3]. A diagnosis is confirmed by histopathological results and immunohistochemical examination of the primary tumor or metastatic lesions. Ultrasound, computed tomography (CT), and magnetic resonance imaging are used for tumor localization, although their use is not widely reported for gastrinoma [4]. Regarding treatment, antisecretory drugs, including proton pump inhibitors (PPIs), have been used for the long-term control of excessive gastric acid secretion in dogs; however, long-term prognoses remain generally poor [1]. Furthermore, available medical therapies targeting the tumor itself are still limited [6,7,8]. To date, no established methods have been reported for monitoring local recurrence or disease progression during long-term post-surgical follow-up. We present here two cases of dogs with gastrinoma. Both cases underwent non-oncologic medical therapy, and serum gastrin concentrations were used as an adjunctive monitoring marker during follow-up. Changes in serum gastrin concentrations were used to support decisions regarding further CT imaging. As for treatment, gastrointestinal signs were managed by administering PPIs, whereas the tumors themselves were treated with surgical therapy. Repeated resections of metastases were performed as needed following resection of the primary tumor. Both dogs survived for approximately 46 months after diagnosis while maintaining good quality of life despite the development of metastatic disease during the clinical course.

## 2. Case Presentation

### 2.1. Case 1

An 8-year-old spayed female Shiba Inu presented at the Hokkaido University Veterinary Teaching Hospital (Sapporo, Japan) for examination regarding weight loss, chronic diarrhea, and progressive anorexia that started 1 month prior. No abnormalities were found during physical examination. Blood test findings revealed mild leukocytosis (18,330/µL; reference range: 5050–16,760/µL) in the complete blood count, and biochemical test results showed a mild electrolyte imbalance and elevated lipase activity (349 U/L; reference range: 10–160 U/L). Chest and abdomen radiography showed no abnormalities. Abdominal ultrasonography revealed a localized, laminar defect in the duodenum and a hypoechogenic mass (18 × 13 mm) in the splenic body. The refractory gastrointestinal symptoms and findings suggestive of a duodenal ulcer raised suspicion for gastrinoma as a differential diagnosis; therefore, fasting serum gastrin concentrations were measured at an external laboratory. Serum gastrin concentrations were measured at an external commercial laboratory (BML, Inc., Tokyo, Japan) by radioimmunoassay with polyethylene glycol separation (RIA-PEG method) using a commercially available assay kit (Gastrin RIA Kit II; TFB Co., Ltd., Tokyo, Japan), for which the laboratory-provided reference interval was 11.9–46.9 pmol/L. Blood samples were collected into serum separator tubes and allowed to clot for 15–30 min at room temperature. Samples were then centrifuged at 3000 rpm for 10 min, and the separated serum was immediately frozen and shipped frozen to the laboratory. All measurements in both dogs were performed at the same laboratory using the same assay method throughout the follow-up period, with no changes in assay methodology. Fluid therapy with Soldem 3 (Terumo Corporation, Tokyo, Japan) was administered on the day of admission to correct electrolyte imbalance. Upper gastrointestinal endoscopy and contrast-enhanced CT of the chest and abdomen were performed under general anesthesia. All subsequent contrast-enhanced CT examinations included the chest and abdomen, with a quadruple-phase protocol for the abdomen. Contrast-enhanced CT examinations were performed using an 80-row multidetector CT scanner (Aquilion Prime, Toshiba Medical Systems Corp., Otawara, Japan). Iohexol (Omnipaque, GE HealthCare, Tokyo, Japan) was administered intravenously through a cephalic vein at a dose of 600 mgI/kg over 20 s. Multiphase CT images were acquired using a bolus-tracking technique. A region of interest was placed in the aorta at the level of the cranial margin of the diaphragm, with a trigger threshold of 200 HU. The early arterial phase was acquired 8 s after triggering, followed by the late arterial (pancreatic) phase 5 s later and the portal phase 20 s thereafter. The delayed phase was acquired 180 s after the start of contrast medium administration. Endoscopy revealed erosions and ulcers extending from the gastric pylorus to the duodenum, and endoscopic biopsies were obtained from the stomach, duodenum, and jejunum. CT showed one jejunal lymph node exhibiting hyper-attenuation compared with other abdominal lymph nodes, although no lesions were visible in the pancreas. On the day of admission (day 0), omeprazole (1 mg/kg; AstraZeneca Pharma Inc., Osaka, Japan) was administered intravenously along with oral sucralfate (1 g every 12 h; Nichi-Iko Pharmaceutical Co., Ltd., Toyama, Japan) as an additional treatment.

**Figure 1:**
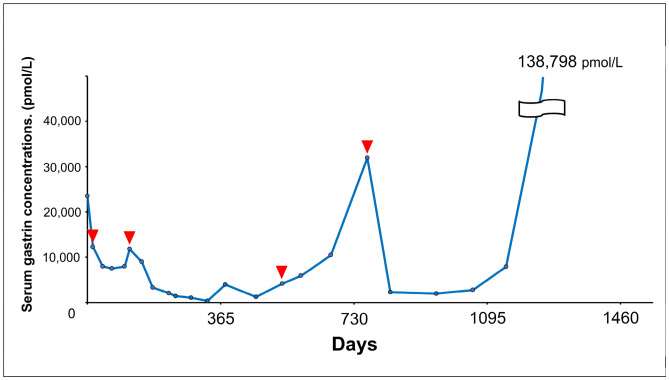
Case 1: Serum gastrin concentrations. Concentrations were measured immediately before each surgery (▼). Surgery was performed four times, on days 31, 141, 540, and 827.

On day, the clinical signs had vanished, and the dog was discharged with a prescription for oral omeprazole (0.7 mg/kg every 24 h) and oral sucralfate (1 g every 12 h). Histopathological examinations did not show abnormalities in the stomach, and suppurative enteritis was diagnosed in the duodenum and jejunum. Serum gastrin concentrations were markedly elevated on day 0 (23,488 pmol/L); therefore, gastrinoma was strongly suspected (Figure 1). Serial serum gastrin concentrations during follow-up are shown in Figure 1, and fasting status, PPI regimens, and serum gastrin concentrations at clinically relevant time points are summarized in Table 1.

On day 31 after initial admission, an exploratory laparotomy was performed. Fentanyl citrate (5 μg/kg; Daiichi Sankyo Company, Ltd., Tokyo, Japan) was administered intravenously as a pre-anesthetic. Anesthesia was induced using intravenous propofol (10 mg/kg). After endotracheal intubation, anesthesia was maintained via mechanical ventilation using isoflurane, pure oxygen (0.5 L/min), and air (0.5 L/min). Intraoperative and postoperative pain was managed using fentanyl (5–20 μg/kg/h) provided as a constant rate infusion. Atropine (20 µg/kg; Mitsubishi Tanabe Pharma Co., Osaka, Japan) was administered intravenously to manage bradycardia during surgery. Dopamine (5.0 µg/kg/min; Teva Takeda Pharma Ltd., Nagoya, Japan) was provided as a constant rate infusion to manage hypotension. The antibiotic cefazolin (20 mg/kg; Nichi-Iko Pharmaceutical Co., Ltd.), was administered intravenously and re-administered every 90–120 min until the end of surgery. No obvious abnormalities were detected in the pancreas, small intestine, or liver upon visual inspection or palpation. Preoperative CT revealed one enlarged lymph node with marked contrast enhancement, and macroscopic observation during surgery revealed two enlarged jejunal lymph nodes adjacent to the pancreas, which were removed. The dog’s postoperative condition was favorable, and the dog was discharged 4 days after surgery, with continuation of omeprazole and sucralfate at the same dosages as before surgery.

Histopathological examination did not show any neoplastic lesions in the resected jejunal lymph nodes. Serum gastrin concentrations had decreased (7986 pmol/L) 10 days after surgery (Figure 1). The dog was in good general condition and did not present with any gastrointestinal symptoms; thus, medical treatment was continued with monthly measurements of serum gastrin concentrations. Throughout the follow-up period, thoracic radiography and abdominal ultrasonography were performed regularly.

The dog occasionally presented with self-limiting vomiting, although her general condition was good and no further weight loss was observed. Given that the serum gastrin concentrations remained high, CT was performed again on day 116 under general anesthesia to search for gastrinoma lesions, revealing marked contrast enhancement in the jejunal lymph node. This lymph node was suspected to be the gastrinoma lesion seen on the CT scan taken on day 1, indicating that it was not resected during surgery. No lesions were detected in the pancreas. The serum gastrin concentrations on day 116 remained elevated (11,759 pmol/L) (Figure 1).

**Figure 2:**
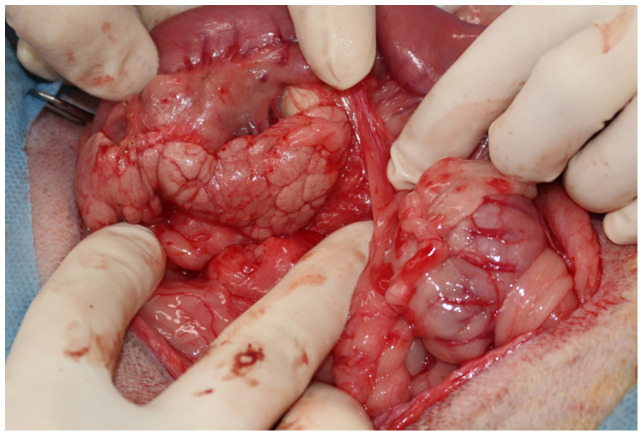
Intraoperative findings at the time of the second surgery in Case 1. Although no abnormalities were observed on computed tomography, partial resection of the pancreas was performed because induration of the right pancreatic lobe was palpable.

**Figure 3:**
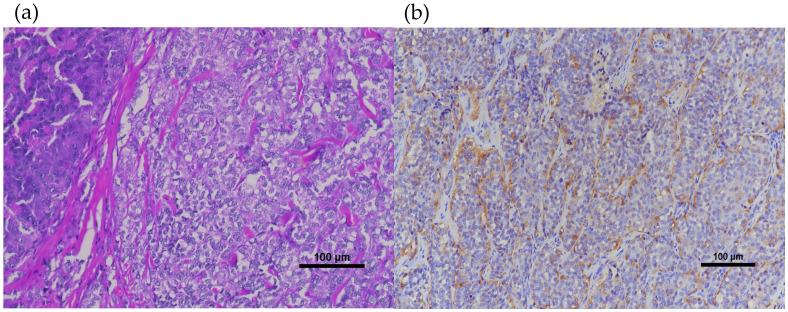
Histopathological and immunohistochemical findings of the resected pancreatic lesion in Case 1. (**a**) The neoplastic cells had abundant, weakly eosinophilic granular cytoplasm and uniform oval nuclei (hematoxylin and eosin staining). (**b**) Immunohistochemical staining showed cytoplasmic immunoreactivity for gastrin in the neoplastic cells. Scale bars = 100 µm.

On day 141, exploratory laparotomy was performed using the same methods used on day 31, and the contrast-enhanced jejunal lymph node was removed. Although no abnormalities were seen in the pancreas on preoperative CT, gross findings revealed a red-tan color and swelling of the margin of the right pancreatic lobe, with palpable induration. The right pancreatic lobe was partially resected using the guillotine technique with 3-0 polydioxanone (Johnson & Johnson K.K., Tokyo, Japan; Figure 2). After the abdomen was closed and postoperative CT confirmed that the contrast-enhanced jejunal lymph node had been removed, the dog was awakened from anesthesia. The resected pancreatic tissue and jejunal lymph node were fixed in 10% neutral buffered formalin and embedded in paraffin wax. Sections (4 µm thick) were prepared and stained with hematoxylin and eosin (HE) for histopathological examination. For immunohistochemistry, sections of the pancreatic mass were subjected to heat-induced antigen retrieval using a microwave oven. Immunohistochemistry was performed using a ready-to-use polyclonal rabbit anti-human gastrin antibody (IR519; Dako, Glostrup, Denmark). The sections were then incubated with Histofine Simple Stain MAX-PO (MULTI) (Nichirei Biosciences, Tokyo, Japan). Antigen–antibody complexes were visualized with 3,3′-diaminobenzidine (DAB) solution (Nichirei Biosciences, Tokyo, Japan), and the sections were counterstained with hematoxylin. Normal canine gastric tissue was used as a positive control and showed appropriate immunoreactivity for gastrin. For the negative control, the primary antibody was replaced with normal rabbit serum (FUJIFILM Wako Pure Chemical Corporation, Osaka, Japan) diluted 1:3000 in antibody diluent and processed under the same conditions. Histopathological examination of the pancreatic mass revealed a poorly demarcated neoplasm composed of atypical cells arranged in lobules separated by fibrovascular stroma. The neoplastic cells had abundant, finely granular, pale eosinophilic cytoplasm and relatively uniform, oval, pale-staining nuclei. Mitotic figures were infrequent. Infiltrative growth of the neoplasm was observed, but no neoplastic cells were identified at the surgical margins. The jejunal lymph node was almost completely replaced by neoplastic cells morphologically similar to those in the pancreatic mass, indicating lymph node metastasis. Immunohistochemically, the neoplastic cells in the pancreatic mass were positive for gastrin, confirming the diagnosis of gastrinoma (Figure 3). Immunohistochemistry was performed only on the primary pancreatic lesion. Metastatic lesions were diagnosed based on their histopathological features, together with the confirmed diagnosis of pancreatic gastrinoma and the clinical course. After histopathological confirmation of the pancreatic gastrinoma, the initial CT images were retrospectively reviewed; however, no corresponding pancreatic lesion could be identified.

On the day after the second surgery, the dog presented with anorexia and vomiting, and blood test findings revealed marked increases in lipase activity (632 U/L) and C-reactive protein level (>20 mg/dL), leading to a diagnosis of acute pancreatitis. The dog received fluid therapy with a glucose-containing electrolyte solution (Soldem 3; Terumo Corp., Tokyo, Japan) and supportive care with maropitant (1 mg/kg) and omeprazole (0.7 mg/kg). Seven days after the second surgery (day 148), the dog was discharged with a prescription for an increased oral omeprazole dose (1.4 mg/kg every 12 h) and the same oral sucralfate dose (1 g every 12 h).

After the second surgery, serum gastrin concentrations were measured every 1–3 months. The dog’s general condition continued to be favorable, without gastrointestinal symptoms, and serum gastrin concentrations showed a downward trend, with the lowest level seen on day 329 (371 pmol/L; Figure 1). The omeprazole dosage was reduced on day 419 and discontinued on day 449. The use of sucralfate hydrate was discontinued on day 284.

On day 534, the dog presented again at our clinic with acute vomiting and anorexia that began 4 days prior, which did not improve after treatment with antiemetics (maropitant) from the primary care veterinarian. Abdominal ultrasonography was not performed on this day because of severe nausea. Upon admission, the dog received intravenous omeprazole (1 mg/kg) and infusion therapy in addition to the antiemetic medication. The dog was able to eat on her own 9 h after omeprazole administration, and the clinical signs disappeared the following day. A marked increase in gastrin concentrations (4187 pmol/L) was observed (Figure 1). Thus, a high possibility of gastrinoma recurrence was considered.

CT was performed on day 538 under general anesthesia. The caudal margin of the right pancreatic lobe showed no differences in contrast enhancement compared to surrounding tissue but demonstrated enlargement with a short axis of 2 cm. No lymph nodes exhibiting abnormal contrast enhancement patterns or enlargement were identified. Recurrence of gastrinoma was suspected at the resection margin of the right lobe of the pancreas from the previous surgery.

An exploratory laparotomy was performed on day 540 (conducted using the same methods as those used on day 31) to partially resect the right pancreatic lobe. The splenic mass identified at initial presentation, which had remained stable in size on follow-up imaging, was also resected. Histopathological examination revealed no neoplastic lesions in the right pancreatic lobe. The splenic mass was diagnosed as a hematoma. The day after surgery, the dog presented with anorexia and vomiting, and blood test results revealed a marked increase in lipase activity (>1000 IU/L) and C-reactive protein level (15 mg/dL), leading to a diagnosis of acute pancreatitis. The dog received electrolyte therapy (Soldem 3) and supportive care with maropitant (1 mg/kg) and omeprazole (1.0 mg/kg). The dog was discharged on day 555 with a prescription for oral omeprazole (1.6 mg/kg every 24 h).

Postoperatively, serum gastrin concentrations did not decrease and they subsequently showed an upward trend. On day 767, gastrin concentrations were higher (31,945 pmol/L) than before the first gastrinoma resection (day 1) (Figure 1). The dog exhibited intermittent gastrointestinal signs but did not lose weight and remained in good general condition. Abdominal ultrasound revealed no abnormalities, and CT on day 786 revealed markedly enhanced lymph nodes in the abdomen near the site where the jejunal lymph node had been removed during the second surgery and markedly enhanced and enlarged anterior mediastinal lymph nodes in the chest.

**Figure 4:**
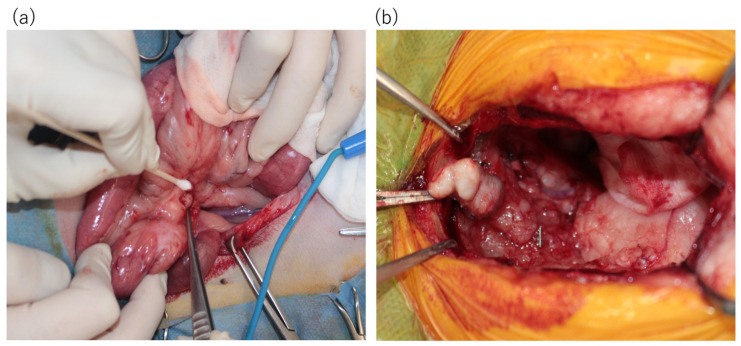
Intraoperative findings at the time of the fourth surgery in Case 1. (**a**) Enlarged intra-abdominal lymph nodes. (**b**) Enlarged mediastinal lymph nodes. Histopathological examination of both lymph nodes confirmed metastatic neuroendocrine tumors considered to be metastases of the pancreatic gastrinoma based on the clinical history.

Exploratory laparotomy and thoracotomy (sternotomy) were performed on day 827 using the same methods as those used on day 31. We resected the intra-abdominal lymph nodes and anterior mediastinal lymph nodes (Figure 4) that were suspected of being metastatic lesions. Histopathological examination revealed that the intra-abdominal and anterior mediastinal lymph nodes were almost completely replaced by neoplastic cells arranged in nests separated by connective tissue stroma. The neoplastic cells had scant, pale eosinophilic cytoplasm and small, round atypical nuclei with mild to moderate anisokaryosis. Mitotic figures were infrequent. Considering the previous diagnosis of pancreatic gastrinoma and the clinical course, these lesions were diagnosed as metastatic gastrinoma.

The dog had a favorable postoperative course and continued the same oral omeprazole dose (1.6 mg/kg every 24 h). The dog was discharged on postoperative day 3 (day 830), at which time serum gastrin concentrations had markedly decreased (2294 pmol/L) (Figure 1).

On day 1169, the dog had a favorable general condition but presented with increasingly frequent anorexia accompanied by intermittent vomiting for the previous month. The oral omeprazole dose was doubled (1.6 mg/kg every 12 h), and a CT scan was scheduled. On day 1245, despite the dog’s generally good condition, CT showed a structure without contrast enhancement that occupied space from the bifurcation of the pulmonary artery to the right pulmonary artery lumen; this lesion was continuous with the right middle tracheobronchial lymph nodes. No lymph nodes showed marked contrast enhancement in the abdomen or chest. Additionally, mild enlargement of both adrenal glands was observed (caudal pole diameter: right, 7.2 mm; L, 8.2 mm). Serum gastrin concentrations on day 1245 were markedly elevated (46,685 pmol/L) (Figure 1). An adrenocorticotropic hormone stimulation test finding showed elevated serum cortisol levels (25.2 μg/dL; reference range: 6–18 μg/dL) 1 h after adrenocorticotropic hormone administration. Oral trilostane (0.8 mg/kg every 12 h) was administered to treat the hyperadrenocorticism.

On day 1331, ultrasonography showed a reduction in the lesion in the right artery, suggesting that this was a thrombus. The remaining lesion was considered a metastatic gastrinoma lesion; however, no other obvious lesions were found in the pulmonary artery. The dog’s general condition was good, and gastrointestinal symptoms were well controlled, although gastrin concentrations were strongly elevated (138,798 pmol/L). A follow-up appointment was scheduled for 2 months later to discuss additional treatment options.

On day 1394, the dog had an acute loss of appetite and underwent symptomatic treatment with the primary care veterinarian, although the dog’s general condition had remained favorable until several days prior. However, that same day, the dog developed respiratory distress and died.

### 2.2. Case 2

A 10-year-old unneutered male Shiba Inu presented at Hokkaido University Veterinary Teaching Hospital for further examination regarding weight loss from chronic vomiting and anorexia of unknown cause that started 2 months prior.

No abnormalities were found during initial physical examination (day 0). Complete blood count revealed leukocytosis (31,530/µL; reference range: 5050–16,760/µL), and biochemical test results indicated elevated lipase activity (926 U/L), without other remarkable abnormalities. Chest and abdomen radiographs showed no abnormalities. Abdominal ultrasonography revealed fluid accumulation from the duodenum to the lumen of the jejunum, with linear hyperechoicity in the mucosal layer of the jejunum. On day 1, Upper gastrointestinal endoscopy and contrast-enhanced CT of the chest and abdomen were performed under general anesthesia, with the same parameters as in Case 1. A region with heterogeneous contrast enhancement in the distal end of the right pancreatic lobe and enlarged jejunal lymph nodes and pancreaticoduodenal lymph nodes were observed (these lymph nodes showed the same level of contrast enhancement as the other lymph nodes). Gross endoscopy findings showed erosion and ulcer lesions from the gastric body to the pylorus and duodenal mucosa and thickening of the pyloric mucosa. Endoscopic biopsies were obtained from the stomach, duodenum, and jejunum. At this time, a lesion was found on the pancreas and gastrinoma was considered a cause of the gastrointestinal signs; fasting serum gastrin concentrations were measured. The dog received intravenous omeprazole (0.7 mg/kg) and subcutaneous maropitant (1 mg/kg) that night. He was able to eat on his own, and the vomiting disappeared. The next day, the dog was discharged with a prescription for oral lansoprazole (1.1 mg/kg every 24 h) and oral sucralfate (1 g every 12 h).

**Figure 5:**
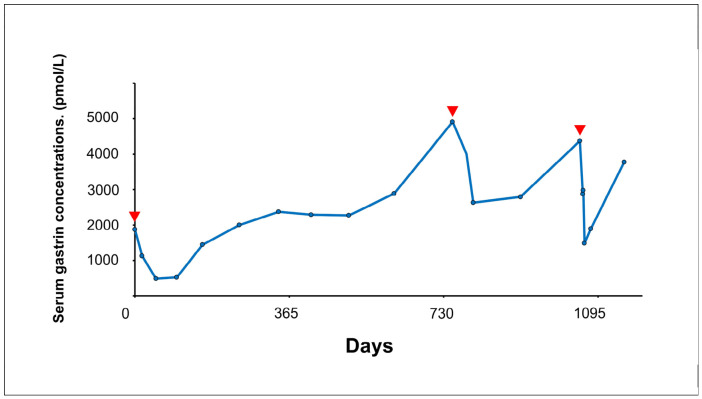
Case 2: Serum gastrin concentrations. Levels were measured immediately before each surgery (▼). Surgery was performed three times, on days 15, 784, and 1057.

Histopathological examinations of resected tissue showed pyloric mucosal hyperplasia in the stomach; lymphangiectasia and suppurative enteritis were diagnosed in the duodenum and jejunum. Serum gastrin concentrations were markedly elevated (1876 pmol/L) (Figure 5 and Table 2). Gastrinoma was strongly suspected.

**Figure 6:**
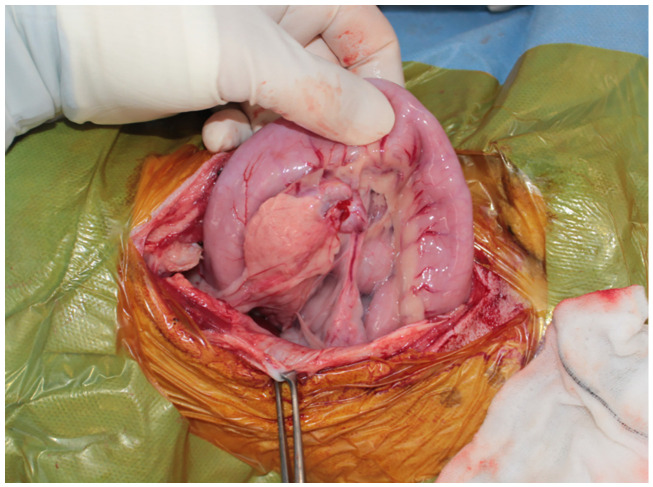
Initial surgery in Case 2. A solid mass lesion was found at the tip of the right lobe of the pancreas.

On day 15, an exploratory laparotomy was performed. Fentanyl (5 μg/kg) was administered intravenously as a pre-anesthetic. Anesthesia was induced using intravenous administration of propofol (10 mg/kg; Mylan Seiyaku, Tokyo, Japan). After endotracheal intubation, anesthesia was maintained with mechanical ventilation using isoflurane, pure oxygen (0.5 L/min), and air (0.5 L/min). Intraoperative and postoperative pain was managed using remifentanil (10–40 μg/kg/h; Ultiva; Janssen Pharmaceutical K.K., Tokyo, Japan) provided as a constant rate infusion. Atropine (20 µg/kg) was administered intravenously to manage bradycardia during surgery. Dopamine (5.0 µg/kg/min) was provided as a constant rate infusion to manage hypotension. The antibiotic cefazolin (20 mg/kg) was administered intravenously and re-administered every 90–120 min until the end of surgery. Partial resection of the right pancreatic lobe using the guillotine technique and resection of the jejunal and pancreaticoduodenal lymph nodes were performed (Figure 6). Histopathological examination of the pancreatic mass revealed an infiltrative neoplasm composed of atypical cells arranged in nests separated by fibrovascular stroma. The neoplastic cells had abundant, granular eosinophilic cytoplasm and round to oval, pale-staining nuclei with mild anisokaryosis. Mitotic figures were occasionally observed. No neoplastic invasion into the surrounding adipose tissue was identified, and no neoplastic cells were identified at the surgical margins. No neoplastic lesions were identified in the jejunal or pancreaticoduodenal lymph nodes. Immunohistochemistry for gastrin was performed on sections of the pancreatic mass using the same protocol as described for Case 1. Immunohistochemistry was performed only on the primary pancreatic lesion. Metastatic lesions were diagnosed based on their histopathological features, together with the confirmed diagnosis of pancreatic gastrinoma and the clinical course. The neoplastic cells in the pancreatic mass were positive for gastrin, confirming the diagnosis of gastrinoma. The dog showed a favorable postoperative course and was discharged 3 days after surgery (day 18) with the same prescription as before surgery for lansoprazole and sucralfate. On day 17 (2 days after surgery), the serum gastrin concentrations had decreased to 1125 pmol/L; on day 50, it had decreased further to 485 pmol/L (Figure 5).

CT was performed 3 months after surgery (day 99) under general anesthesia. The dog had vomited once during the 3 months following surgery and was in good general condition. However, detailed ultrasonography was difficult due to aggressive behavior. After consultation with the owner, CT was performed under general anesthesia. No swelling of the intra-abdominal lymph nodes was observed, and there were no lesions showing contrast enhancement in any of the phases. The serum gastrin concentration was 522 pmol/L on day 99.

The serum gastrin concentration increased to 1441 pmol/L on day 160 (Figure 5). The dog’s general condition was good, with no gastrointestinal signs observed. Although serum gastrin concentrations remained elevated, it was decided in consultation with the owner to continue omeprazole at the same dose while monitoring serum gastrin concentrations and the dog’s clinical condition. On day 613, abdominal ultrasonography was not performed because the dog had clearly demonstrated intolerance to restraint and the owner wished to minimize clinic time. The owner and care team agreed that no further ultrasound examinations would be performed and that only serum gastrin measurements and chest radiography would be conducted at follow-up visits.

On day 751, the dog’s general condition was good, with no observed gastrointestinal signs. As in previous follow-ups, blood samples were collected for gastrin measurement and chest radiography was performed. The dog was discharged after the examination while awaiting the externally processed serum gastrin results. The dog remained fasted while at the hospital, with a long wait time (~6 h) until examination. The serum gastrin concentrations showed a markedly elevated level (4911 pmol/L) (Figure 5), and the owner was contacted by telephone. The owner reported that no gastrointestinal signs had been observed up to the day of examination (day 751); however, gastrointestinal symptoms, including vomiting and anorexia, developed 1 day later (day 752) and progressively worsened despite treatment with an antiemetic (maropitant) administered by the primary care veterinarian. The dog was admitted to our clinic on day 760 and received intravenous omeprazole (2 mg/kg) with fluid infusion therapy. The clinical signs disappeared the following day.

CT was performed 3 days after admission (day 763) under general anesthesia. In the arterial phase, marked contrast enhancement was observed in the left and right hepatic, splenic, and jejunal lymph nodes and greater omentum. These lymph nodes were more enlarged than on the previous CT examination, suggesting they were metastatic gastrinoma lesions.

**Figure 7:**
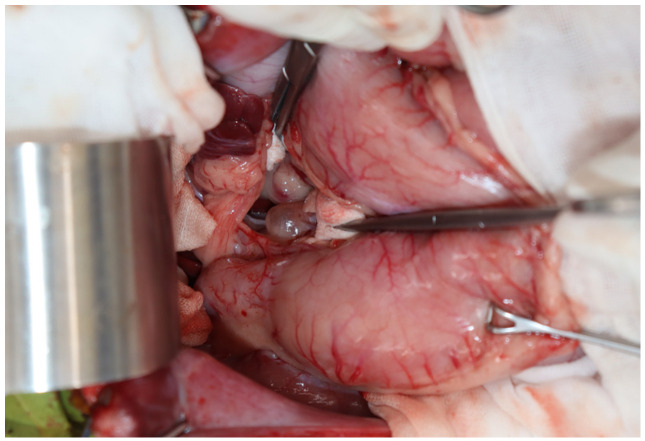
Macroscopic findings during the second surgery in Case 2 showing metastatic liver lymph nodes.

**Figure 8:**
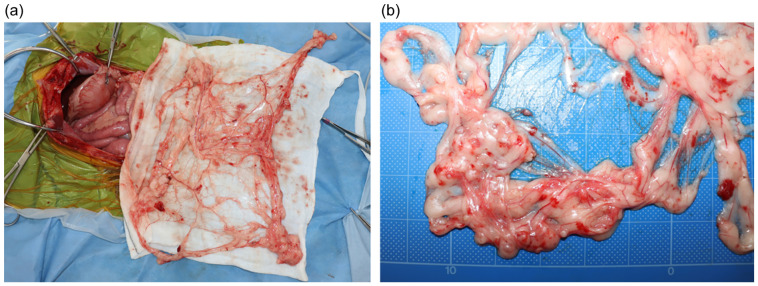
Macroscopic findings from the second surgery in Case 2. (**a**) Overall view of the intraperitoneal lymph nodes and omentectomy. (**b**) A close-up photograph of the greater omentum. Extensive disseminated lesions were observed in the greater omentum.

On day 784, an exploratory laparotomy was performed using the same methods as those used on day 15. The left and right hepatic, splenic, and jejunal lymph nodes and greater omentum were resected (Figure 7 and Figure 8). Histopathological examination revealed multifocal neoplastic lesions in the omentum and hepatic lymph nodes. The lesions were lobulated and separated by thin connective tissue septa and were composed of neoplastic cells with abundant, finely granular, pale eosinophilic cytoplasm and round, pale-staining nuclei. Mitotic figures were rare. Considering the history of pancreatic gastrinoma, these lesions were diagnosed as metastatic gastrinoma. No neoplastic lesions were identified in the jejunal or splenic lymph nodes.

The postoperative course was favorable, and the dog was discharged 3 days after surgery (day 787) with an increased prescription for oral lansoprazole (2 mg/kg every 24 h). Sixteen days after surgery, serum gastrin concentrations had decreased (2632 pmol/L) and the oral lansoprazole dose was reduced to the original dose (1 mg/kg every 24 h). The gastrointestinal signs disappeared, and the dog had a favorable general condition.

**Figure 9:**
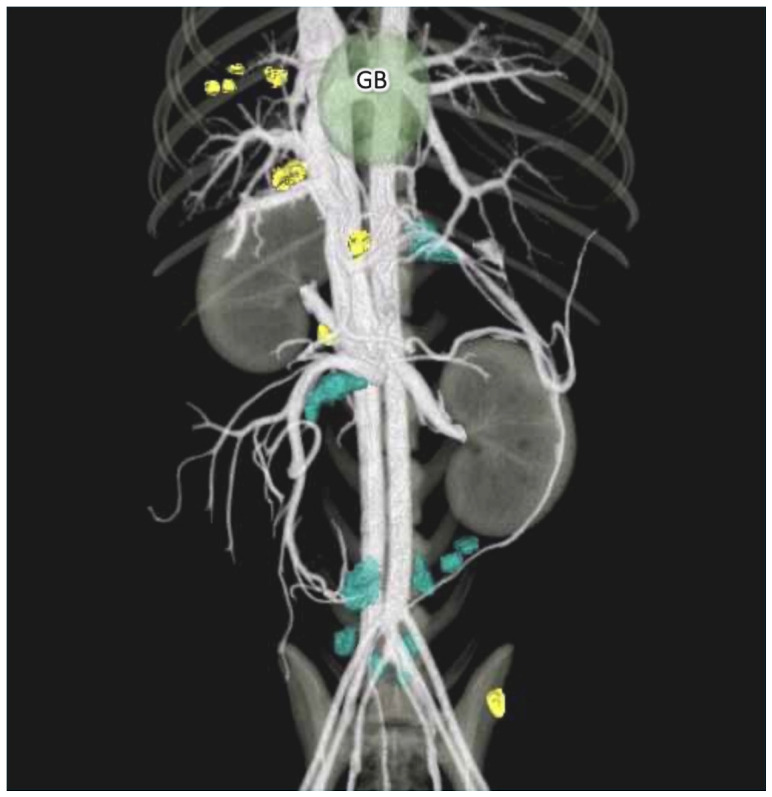
Three-dimensional reconstruction from CT images in Case 2. Preoperatively suspected metastatic lesions, including markedly enhanced intra-abdominal lymph nodes and nodular lesions, are shown in yellow. Enlarged intra-abdominal lymph nodes without marked enhancement are shown in light blue. GB: gall bladder.

**Figure 10:**
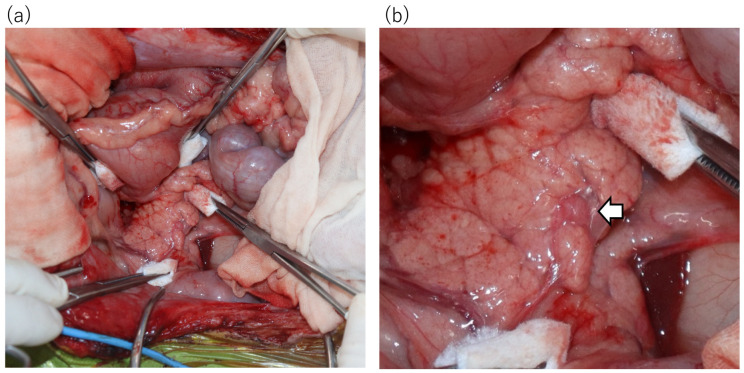
Macroscopic findings from the third surgery in Case 2. (**a**) Photograph of the nodular lesion in the pancreas that was not detected on preoperative computed tomography. (**b**) Close-up photograph of the nodular lesion. The arrow indicates the nodular lesion. The nodular lesion was histopathologically diagnosed as gastrinoma.

**Figure 11:**
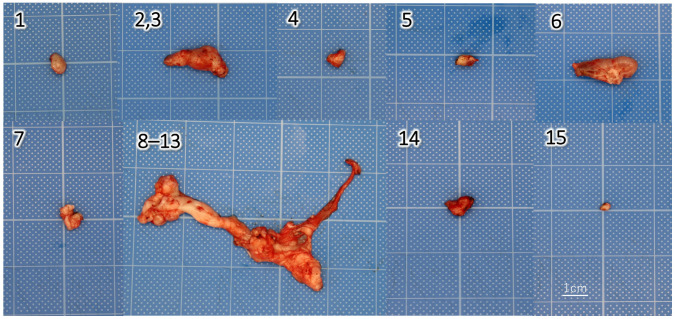
Specimens removed during the third surgery in Case 2. Two nodular lesions in the pancreas, 12 lymph nodes, and one ganglion were removed. The two pancreatic nodular lesions, the right hepatic lymph node lesion, and the lymph nodes of the lesser omentum were histopathologically confirmed to be metastatic gastrinoma lesions. Tumorous lesions were not found in the remaining four specimens. 1: The nodular lesion observed near the deep inguinal ring, 2, 3: Jejunal lymph nodes, 4: Right hepatic lymph node, 5: ganglion, 6: Splenic lymph node, 7, 8–13: Lymph nodes within the lesser omentum, 14, 15: The two nodular lesions in the pancreas.

On day 1031, the dog’s general condition was good and the owner noted that vomiting occasionally occurred when the dog was hungry. Examination showed elevated gastrin concentrations (4567 pmol/L) (Figure 5). On day 1052, CT was performed under general anesthesia and contrast-enhanced lesions were observed in the arterial phase in the right hepatic lobe, lesser omentum, and pancreatic body, which were considered metastatic lesions from the gastrinoma. Additionally, a nodular lesion with marked contrast enhancement was observed near the deep inguinal ring. Although lymph nodes are not normally present in this location, metastasis from a gastrinoma was suspected. Additional nodules were observed with marked contrast enhancement symmetrically on both sides, encircling the base of the celiac artery, although it was suspected to be a ganglion. The splenic and jejunal lymph nodes were enlarged compared to observations on the previous CT, although no marked contrast enhancement was observed. Furthermore, because the splenic lymph nodes were located upstream of the pancreatic tumor along the lymphatic drainage pathway, they were considered unlikely to represent lymphatic metastases from the gastrinoma (Figure 9). On day 1057, an exploratory laparotomy was performed using the same methods as those used on day 15. Lymph nodes showing enlargement on preoperative CT were resected even if they did not demonstrate marked contrast enhancement, as the possibility of metastasis could not be ruled out. Since we could not rule out the possibility that the suspected ganglion nodule was also a metastatic lesion, we resected one of the two. Additionally, a nodular lesion in the pancreatic body detected on preoperative CT was resected. Although not visible on preoperative CT, a pink, indurated nodular lesion was identified macroscopically (Figure 10) and this was also resected with a narrow margin. Therefore, 15 surgical specimens were removed in total (Figure 11). Histopathological examination revealed neoplastic lesions in the two pancreatic lesions and in nine lymph nodes located downstream of the lymphatic flow from the pancreas. The neoplasms were lobulated and separated by thin connective tissue septa and consisted of neoplastic cells with abundant, finely granular, pale eosinophilic cytoplasm and round, pale-staining nuclei. Mitotic figures were rare. In the metastatic lymph nodes, the normal architecture was almost completely effaced by neoplastic cells morphologically similar to those in the pancreatic lesions. Based on these findings and the history of pancreatic gastrinoma, the lesions were diagnosed as metastatic gastrinoma. No neoplastic lesions were identified in the three lymph nodes located upstream of the pancreas. The nodular lesion near the deep inguinal ring was diagnosed as a metastatic lesion and was considered a site of intra-abdominal dissemination. The suspected ganglion was histopathologically confirmed to be a ganglion without neoplastic lesions. Neoplastic cells were identified at the surgical margin of the pancreatic body lesion.

The postoperative course was favorable, and the dog was discharged 3 days after surgery (day 1060). Six days after surgery (day 1063), the gastrin concentrations had decreased (1484 pmol/L) (Figure 5).

On day 1157, the serum gastrin concentrations were elevated (3781 pmol/L) (Figure 5). However, the owner decided to continue pharmacological therapy with omeprazole alone, without any further examinations or surgeries. The gastrin concentrations were not measured again, and the dog died on day 1397. In a telephone follow-up, the owner stated that the dog’s general condition had been favorable until 2 weeks before his death.

## 3. Discussion

In the two dogs described herein, serum gastrin concentrations were measured during their first visit while fasted and not receiving any antisecretory drugs. However, the serum gastrin concentrations at later regular checkups were measured without pausing PPI treatment and without fasting. This was due to concerns that pausing PPI treatment or fasting would cause various gastrointestinal symptoms such as severe gastroduodenal ulcers to recur [9]. In humans, the use of PPIs increases serum gastrin concentrations; therefore, their administration should be paused during diagnostic evaluations for gastrinoma because of overlapping gastrin concentrations between patients with gastrinoma and controls [10]. However, in recent years, proposals to assess the pros and cons of medication cessation on a case-by-case basis have been reported owing to possible recurrence of severe gastrointestinal ulcers after PPI cessation [9,11]. The dog in Case 2 developed a recurrence of gastrointestinal signs after examination on day 751. However, considering that no gastrointestinal signs were observed until the examination day, fasting and stress during the examination and waiting period may have triggered the recurrence of the gastrointestinal signs.

In dogs, whether PPI administration or feeding leads to a misdiagnosis of gastrinoma is unknown [12], although previous studies have shown that increases in serum gastrin concentrations associated with PPI administration or feeding are generally modest [5,13]. However, the effect of long-term PPI administration on serum gastrin concentrations in dogs remains unclear, which limits the interpretation of serial serum gastrin concentrations in the present cases. In dogs, no specific cutoff value has been set for gastrinoma diagnosis, and the lowest reported serum gastrin concentration was 72 pg/mL, as reported in the original publication [14]. Thus, careful evaluations should be made, especially regarding pausing PPI use and fasting at the time of diagnosis. In both dogs, serum gastrin concentrations remained markedly elevated even after all gross lesions had been removed. Although PPI administration and feeding may have contributed to these elevations, unrecognized residual disease, early recurrence, or metastasis could not be excluded. Therefore, recurrence and metastasis were assessed based on serial changes in serum gastrin concentrations together with imaging findings rather than on absolute serum gastrin concentrations alone.

Both dogs showed marked drops in serum gastrin concentrations after gastrinoma resection. In Case 1, however, no lesions were resected during the first surgery, although the gastrin concentrations dropped substantially thereafter. The reason for this is unclear. The other surgeries were followed by drops in serum gastrin concentrations after gastrinoma lesion resection; however, there was no decrease when lesions could not be removed. Serum gastrin concentrations were the lowest after each surgery and tended to increase as the number of surgeries increased, suggesting that previously unrecognized lesions had increased and that a gradual increase in residual lesions occurred even after all the gross lesions had been resected. In particular, the pancreatic lesions that could not be detected on imaging tests were removed based on intraoperative decisions in both dogs. In retrospect, the two pancreatic lesions found during the third surgery in Case 2 may have been overlooked in the second surgery. Despite the disease progression after the third surgery, we were able to reduce the number of residual lesions compared to after the second surgery; therefore, this may have been the reason for the larger decreases in serum gastrin concentrations after the third surgery than after the second surgery. In these two cases, serial changes in serum gastrin concentrations may have reflected changes in residual tumor burden, although this relationship could not be established. For humans, international guidelines indicate that, after a definitive diagnosis of gastrinomas is made based on hypergastrinemia and excessive gastric acid secretion, detailed imaging diagnostics should confirm the lesion location before surgical treatment. However, some lesions remain undetected by imaging, raising concerns that patients with imaging-negative gastrinoma may experience delayed treatment initiation, leading to disease progression. Norton et al. reported that, in 98% of patients with Zollinger–Ellison syndrome with no lesions detected on imaging, lesions were found intraoperatively, primarily in the duodenum and pancreas [15]. In Case 1, surgery was performed on day 540 because tumor recurrence was suspected based on increased serum gastrin concentrations and imaging findings; however, histopathological examination did not confirm neoplasia, and serum gastrin concentrations did not decrease after surgery. This episode may represent a false-positive scenario, although the possibility that the responsible lesion was not identified and resected cannot be excluded. Thus, increased serum gastrin concentrations and suspicious imaging findings do not necessarily lead to successful identification and resection of the responsible lesion, highlighting a limitation of this monitoring approach when used to guide surgical intervention. Another limitation is that, although analytical validation of a commercially available human radioimmunoassay for serum gastrin measurement in dogs has been reported, the specific assay used in the present cases has not been formally validated for use in dogs. This limitation should be considered when interpreting the serum gastrin concentrations. The findings in these two cases suggest that careful intraoperative macroscopic examination and palpation may be important because some gastrinoma lesions may not be detected on CT. In Case 1, thoracic radiography and abdominal ultrasonography were performed regularly throughout the follow-up period; however, these examinations did not detect the lesions subsequently identified on contrast-enhanced CT. In Case 2, routine abdominal ultrasonography was discontinued after day 613 because of the dog’s intolerance to restraint and the owner’s preference to minimize clinic time, and follow-up was primarily based on serial serum gastrin measurements and chest radiography. Contrast-enhanced CT was performed when recurrent or metastatic disease was suspected and was used for further evaluation and surgical planning. After the first surgery in each dog, the follow-up strategy consisted of serial monitoring of serum gastrin concentrations, performing CT when the concentrations increased markedly, and planning further surgery based on these findings. Repeat surgeries were performed with therapeutic intent to resect suspected recurrent or metastatic lesions rather than to investigate the pattern of tumor progression. Decisions regarding repeat surgery were made on a case-by-case basis, considering serial serum gastrin concentrations, clinical signs, and CT findings. In Case 1, serum gastrin concentrations remained high after the first surgery, prompting repeat CT examination, which revealed that the jejunal lymph node suspected to be a gastrinoma lesion on the initial CT had not been removed during the first surgery. This finding prompted the second surgery. During surgery, induration of the right pancreatic lobe was identified by palpation, and partial pancreatectomy was also performed. Subsequent surgeries in Case 1 and repeat surgeries in Case 2 were performed when lesions suspected to represent recurrent or metastatic disease were identified on CT. However, it remains unclear what degree of increase in serum gastrin concentration should be considered clinically significant for disease progression. Therefore, no predefined serum gastrin cut-off value was used to guide repeat imaging or surgical intervention. Instead, clinical decisions were based on the overall assessment of serial serum gastrin concentrations together with clinical signs and diagnostic imaging findings.

Neuroendocrine tumors, including gastrinoma, generally grow slowly [16], and serum gastrin concentrations might therefore be expected to increase gradually with tumor progression. However, both dogs herein showed rapid increases in serum gastrin concentrations after periods of little change. The lack of substantial changes in serum gastrin concentrations during these periods did not necessarily indicate an absence of tumor growth, and the reasons for these trends remain unclear. These observations suggest that serial serum gastrin measurements may provide adjunctive information during postoperative monitoring; however, fasting serum gastrin concentrations alone may not fully reflect disease activity as secretin provocative testing has been reported to become positive before an increase in fasting serum gastrin concentrations [17].

In humans, oncologic medical therapy is administered for advanced/metastatic gastrinomas, with evidence of efficacy for octreotide, the multitargeted tyrosine kinase inhibitor sunitinib, and streptozocin [1]. In dogs, reports of oncologic medical therapy for gastrinomas are limited. While octreotide administration has been reported to reduce serum gastrin concentrations, no clear tumor shrinkage has been demonstrated and it remains unclear whether it improves prognosis. In recent years, the multitargeted tyrosine kinase inhibitor toceranib has been reported to be effective against various neuroendocrine tumors in dogs [18,19,20], including insulinomas, and may become a treatment option for gastrinomas. Toceranib was not administered in either dog. In Case 1, after the fourth surgery, we planned to discuss nonsurgical treatment options, including toceranib and radiation therapy, with the owner because of the pulmonary artery lesion. However, the dog died before the next scheduled follow-up visit. In Case 2, after the third surgery, the owner declined further blood testing and requested continued PPI therapy alone, and no additional antitumor treatment was administered. In dogs with gastrinoma, it remains unclear whether palliative surgery enhances the efficacy of medical therapy or improves prognosis compared with medical therapy alone. In humans, gastrinoma exhibits a lymph node metastasis rate of approximately 50%. When distant metastasis is absent, resection of the primary tumor and affected lymph nodes has been reported to reduce the risk of liver metastasis, which is one of the main determinants of prognosis, and to prolong disease-free survival [9,21]. In addition, although the role of palliative surgery for advanced metastatic lesions and extensive infiltrating lesions is not well established, surgical resection is generally considered when at least 80% of the tumor burden is resectable [9]. In the present cases, surgery was the only treatment directed at the tumors, and both dogs survived for 46 months after diagnosis while maintaining a good quality of life despite the development of metastatic disease during the clinical course. Although the contribution of surgery to survival cannot be determined from these two cases, the long-term survival observed despite metastatic disease during the clinical course suggests that metastatic disease alone may not preclude consideration of surgical treatment in dogs with gastrinoma.

In the present cases, most gross lesions identified by diagnostic imaging and during surgery were resected, and no metastases to solid organs were detected. The metastatic lesions were mainly confined to the regional lymph nodes, which were amenable to surgical resection. In addition, the primary tumors in both dogs were located in the right pancreatic lobe, which may have facilitated resection with clear margins without serious complications. These anatomical and disease-related characteristics may have contributed to the feasibility of repeated surgical intervention and should be considered when interpreting the long-term outcomes observed in these two cases.

The limitations of these case reports include possible confounding effects of PPI on serum gastrin concentrations, lack of specific and consistent threshold of gastrin concentrations to consider additional surgery, and the lack of necropsy at the time of death. Neither dog underwent postmortem examination; therefore, the final extent of disease progression and the definitive cause of death could not be determined. This represents a limitation of the present report.

## 4. Conclusions

In the present cases, serum gastrin concentrations increased with disease progression and decreased after resection of gastrinoma lesions, suggesting that serial serum gastrin measurements may serve as an indicator of disease activity. Serial serum gastrin measurements, together with diagnostic imaging and clinical signs, may provide adjunctive information when considering further imaging or surgical intervention. In the present cases, careful inspection and palpation during surgery contributed to the identification of lesions that were not detected by preoperative CT. In the present cases, long-term survival was observed despite the development of metastatic disease during the clinical course, suggesting that metastatic disease alone may not preclude consideration of surgical treatment in dogs with gastrinoma.

## Figures and Tables

**Table 1:** Serum gastrin concentrations, PPI treatment, and fasting status at clinically relevant time points in Case 1. Note: PPI regimens had remained unchanged for at least 1 month before each serum gastrin measurement; when no PPI was administered, the dog had been off PPI treatment for at least 1 month before sampling.

	Day	Fasting	PPI Regimen (mg/kg)	Serum Gastrin Concentrations (pmol/L)
Initial presentation	0	Yes	No PPI	23,488
Before first surgery	15	No	1 mg/kg, SID	12,449
Postoperative nadir after the first surgery	67	No	0.7 mg/kg, SID	7533
Before second surgery	116	No	0.7 mg/kg, SID	11,759
Postoperative nadir after the second surgery	329	No	1.4 mg/kg, SID	371
Before third surgery	534	No	Off PPI	4187
Postoperative nadir after the third surgery	585	No	1.6 mg/kg, SID	5937
Before fourth surgery	767	No	1.6 mg/kg, SID	31,945
Postoperative nadir after the fourth surgery	956	No	1.6 mg/kg, SID	1991
Final follow-up	1340	No	1.6 mg/kg, BID	138,798

**Table 2:** Serum gastrin concentrations, PPI treatment, and fasting status at clinically relevant time points in Case 2. Note: PPI regimens had remained unchanged for at least 1 month before each serum gastrin measurement; when no PPI was administered, the dog had been off PPI treatment for at least 1 month before sampling.

	Day	Fasting	PPI Regimen (mg/kg)	Serum Gastrin Concentrations (pmol/L)
Initial presentation/ Before first surgery	0	Yes	No PPI	1876
Postoperative nadir after the first surgery	50	No	1.1 mg/kg, SID	485
Before second surgery	751	No	1.0 mg/kg, SID	4911
Postoperative nadir after the second surgery	800	No	1.0 mg/kg, BID	2632
Before third surgery	1031	No	1.0 mg/kg, SID	4567
Postoperative nadir after the third surgery	1063	No	1.3 mg/kg, SID	1484
Final follow-up	1157	No	1.4 mg/kg, SID	3781

## Data Availability

The original contributions presented in this study are included in the article. Further inquiries can be directed to the corresponding author.

## Abbreviations

The following abbreviations are used in this manuscript:

CT	Computed tomography
PPI	Proton pump inhibitor
URL	Upper reference limit
